# Dyspareunia and Biomarkers: A Case Study of Sexual Dysfunction in Moderate Endometriosis

**DOI:** 10.3390/ijms26010162

**Published:** 2024-12-27

**Authors:** Ionel Daniel Nati, Razvan Ciortea, Andrei Malutan, Mihaela Oancea, Cristian Iuhas, Carmen Bucuri, Maria Roman, Cristina Ormindean, Leon Gombos, Dan Mihu

**Affiliations:** 1 2nd Department of Obstetric and Ginecology, “Iuliu Hatieganu” University of Medicine and Pharmacy, 400610 Cluj-Napoca, Romania; ionel.nati@umfcluj.ro (I.D.N.); rciortear@umfcluj.ro (R.C.); oancead@umfcluj.ro (M.O.); iuhas.cristiann@umfcluj.ro (C.I.); mromanm@umfcluj.ro (M.R.); cormindeanc@umfcluj.ro (C.O.); danmihud@umfcluj.ro (D.M.); 2 Emergency Military Clinical Hospital “Dr Constantin Papilian”, 400610 Cluj-Napoca, Romania; 3 Faculty of Physical Education and Sport, Bogdan-Vodă University of Cluj Napoca, 400394 Cluj-Napoca, Romania

**Keywords:** endometriosis, dyspareunia, sexual function, lubrication

## Abstract

Endometriosis, a chronic hormone-dependent condition affecting 10% of women globally, impacts pelvic organs and occasionally distant sites, causing pain, infertility, and sexual dysfunction. Biomarkers such as IL-8, IL-10, and BDNF influence inflammation, nerve sensitization, and pain. This study investigates their relationship with sexual quality of life, focusing on dyspareunia and related dysfunctions, as assessed using the Female Sexual Function Index (FSFI). Dyspareunia, a prominent symptom of endometriosis, is linked to lower FSFI scores in domains such as desire (mean 3.38), satisfaction (mean 3.28), and pain (mean 3.07). Elevated IL-8 tissue levels negatively correlated with desire (r = −0.649, *p* < 0.05) and satisfaction (r = −0.813, *p* < 0.01). Similarly, higher BDNF tissue levels were associated with increased pain (r = −0.435, *p* < 0.01) and reduced satisfaction (r = −0.252, *p* < 0.05). Patient factors such as higher endometriosis severity scores (mean 26.3, *p* < 0.05) and surgical history correlated with lower desire and satisfaction. Conversely, physical activity improved pain scores (*p* < 0.01) and enhanced desire and lubrication (*p* < 0.05), likely through reduced inflammation and better circulation. These findings highlight the complex interplay between biomarkers, individual factors, and sexual dysfunction in endometriosis, underscoring the need for personalized therapeutic approaches.

## 1. Introduction

Endometriosis is a chronic disease characterized by the presence of endometrial tissue, consisting of endometrial stroma and glands, outside the uterine cavity. It is a hormone-dependent pathology, with most cases occurring in patients of reproductive age, with premenarchal or postmenopausal cases being an exception. The global incidence is around 10%, with an occurrence of almost 50% in patients with chronic pelvic pain and/or infertility [1,2,3,4].

There are several theories regarding the origin of endometriotic lesions, with some of the most compelling being retrograde menstruation, immune deficiency, and hematogenous or lymphatic dissemination. The location of endometriotic lesions can be explained through these theories, with the pelvic organs, such as ovaries, fallopian tubes, uterus, and uterosacral ligaments, being most commonly affected. However, atypical lesions have also been described, such as those on diaphragm, lungs, or other distant organs [5,6,7,8,9,10,11].

The main symptom of endometriosis is pelvic pain in various forms: dysmenorrhea, dyspareunia, chronic pelvic pain, dysuria when the urinary tract is involved, or dyschezia when the intestines are affected. Sometimes, the patient is clinically asymptomatic, with endometriosis being diagnosed during a routine gynecological exam or when investigating infertility [12,13,14,15]. Diagnosis poses a challenge for clinicians given the wide range of possible symptoms and atypical forms of endometriosis. There is no curative treatment; medical management is mainly focused on relieving the patient’s symptoms, improving quality of life, or treating infertility. Treatment options include analgesics, hormonal therapy, minimally invasive surgery for conservative and reconstructive purposes, and radical surgery when necessary [16,17,18,19,20,21,22,23].

Given the chronic nature of endometriosis, its impact on women’s quality of life can be significant, not only affecting the patient’s physical and psychological health, but also her interpersonal relationships. Defining a patient’s quality of life is challenging due to the subjective nature of the term, as it is influenced by socioeconomic status, social belonging, or religious beliefs. It is often described as a sense of well-being, health, comfort, and happiness experienced by an individual. From a medical perspective, it is defined by how a specific pathology affects a patient and is often quantified through questionnaires [24,25,26,27,28,29,30].

Sexual quality of life is closely linked to physical health, psychological well-being, femininity, and relationships. It can be affected by medical conditions as well as physical, psychological, and sociocultural factors. Without a doubt, given the nature of the lesions and associated symptoms, endometriosis can negatively impact women’s sexual lives. This impairment results not only from experience of painful intercourse, but also from a much wider range of sexual dysfunctions, such as reduced sexual desire, arousal, and orgasm. These disruptions may be rooted in fear and anticipation of pain, stemming from repeated painful sexual experiences. Moreover, the molecular mechanisms that may explain these sexual dysfunctions are not fully understood [31,32].

### 1.1. Pelvic Pain and Biomarkers

Macrophages, activated T lymphocytes, and endometriotic lesions themselves play a key role in releasing growth factors and pro-inflammatory or angiogenic cytokines. These substances contribute to the progression of endometriotic lesions, expansion of nerve fiber networks, and development of chronic pelvic pain:

- IL-8 (interleukin 8), a potent α-chemokine with chemotactic and angiogenic properties, is primarily produced by peripheral macrophages and is found in high concentrations in the peritoneal fluid of women with endometriosis. Besides its chemotactic and granulocyte-activating roles, IL-8 promotes the proliferation of endometrial cells.
- IL-10 (interleukin 10), secreted abundantly by macrophages, suppresses T-cell activation and decreases the expression of co-stimulatory molecules like CD-80 and CD-86, as well as indoleamine 2,3-dioxygenase. Additionally, macrophages produce IL-15 (interleukin 15), which acts as a chemoattractant for uNK (uterine natural killer) cells while simultaneously dampening their cytotoxic function.
- Nerve growth factors (NGFs), notably BDNF (Brain-Derived Neurotrophic Factor), are abnormally produced by activated macrophages, mast cells, NK cells, and leukocytes within endometriotic lesions located near sensitive nerve fibers or in peritoneal fluid. These factors sensitize and activate nerve endings, triggering a cycle of nociceptor sensitization, focal neoneurogenesis, and nerve activation, which ultimately results in hyperalgesia.

These biomarkers, along with many others, are considered to be involved in the development of endometriosis lesions and onset of symptoms. In the present study, our focus is directed towards these three described biomarkers [33,34,35].

### 1.2. Objectives

This study aims to investigate the possible relationship between sexual quality of life in patients affected by endometriosis and a series of biomarkers: interleukin 8 (IL-8), interleukin 10 (IL-10), and Brain-Derived Neurotrophic Factor (BDNF).

Dyspareunia in patients with endometriosis may be associated with IL-8, IL-10, and BDNF, as these biomarkers contribute to inflammatory and neurogenic processes that heighten pain sensitivity. IL-8 is known to attract immune cells and promote angiogenesis, creating an inflammatory environment that can increase nerve fiber density around endometriotic lesions. IL-10, though typically anti-inflammatory, may alter immune responses in ways that support chronic inflammation in endometriosis. Meanwhile, BDNF promotes nerve growth and sensitization, intensifying the response of sensory nerve fibers in affected areas. Together, these effects can exacerbate the pain response during intercourse, making dyspareunia a common symptom for patients with endometriosis.

## 2. Results

### 2.1. Patient Characteristics

Following the application of inclusion and exclusion criteria, a study group of 46 patients was formed. In all cases, diagnosis of endometriosis was confirmed via histopathological examination of excised tissue. The mean age of the patients was 28.25 years, with a standard deviation of 7.14. The severity of endometriosis was evaluated using rASRM classification, with an average score of 26. The majority of cases were classified as stage 3 (38 cases), followed by stage 2 (5 cases) and stage 4 (3 cases). The patients’ body mass index (BMI) was also assessed, revealing an average value of 25.31.

### 2.2. Results of the FSFI Questionnaire Applied to Patients

Regarding the FSFI questionnaire, the data are summarized in Table 1. Minimum and maximum values obtained, average values, and standard deviation are presented. We observe the variability of these results, which indicates and supports the fact that endometriosis affects all primary components of sexuality and not just the pain component. The lowest scores were obtained in the pain domain and arousal (3.07 and 3.08), with these also having the lowest maximum values (4.4 and 4.2, respectively). The average total FSFI score was 19.36, with a minimum of 10.7 and a maximum of 25.9.

### 2.3. Expression of Biomarkers at Serum and Tissue Levels

The expression of studied biomarkers is shown in Table 2. The average values of these biomarkers at the serum and tissue levels, as well as standard deviations, are displayed. For IL-8 and IL-10, we observe higher expression at the tissue level compared to their serum levels. In contrast, BDNF shows greater expression in serum than in tissue.

The results of associated factors taken into consideration that may influence the FSFI score are presented in Table 3. The use of hormonal treatment was assigned a value of 1, while the absence of hormonal treatment was assigned a value of 0. In our group, 28 patients were undergoing some form of hormonal treatment (progestins, combined oral contraceptives, or a long-acting progesterone-releasing intrauterine device). The endometriosis severity score showed an average of 26.3, with a minimum of 8 points and a maximum of 40 points. A history of surgical interventions for endometriosis was assigned a value of 1, while the absence of such a history was assigned a value of 0, with 16 patients having a positive surgical history.

Correlations between FSFI components, studied biomarkers, and other factors that can influence the quality of sexual life are presented in Table 4. We note statistically significant correlations for tissue values of IL-8 and BDNF with the desire, satisfaction, and pain domains. The arousal and orgasm domains showed no correlation with the investigated biomarkers and factors. The lubrication domain only correlated with independent factors of hormonal treatment (use of hormonal therapy) and age. Pain and satisfaction domains had the most correlations with investigated factors. Regarding pain, statistically significant correlations were found with tissue levels of IL-8, tissue levels of BDNF, endometriosis severity, and history of surgical interventions. Pain scores were improved if the patient was on hormonal treatment or engaged in regular physical activity, with these associations being statistically significant in a positive way. Sexual satisfaction is negatively influenced by tissue levels of IL-8, both serum and tissue levels of BDNF, endometriosis severity, and history of surgical interventions.

### 2.4. Graphic Analysis of Significant Statistical Correlations Between Biomarkers, Individual Factors and FSFI Components

**Figure 1:**
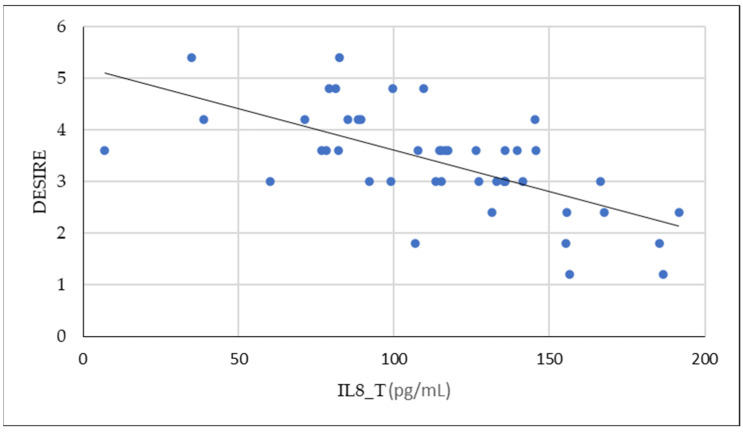
Negative association between IL8_T and desire values (Pearson = −0.649).

In our analysis, we found a negative correlation between IL-8 tissue values and desire. These data are shown graphically in Figure 1.

**Figure 2:**
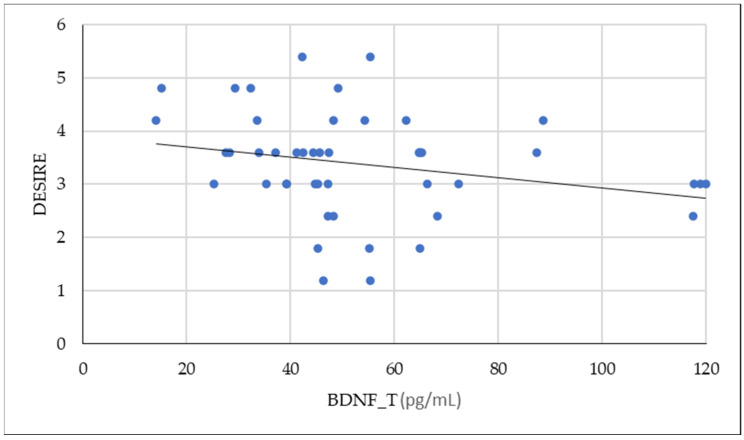
Negative association between BDNF_T values and desire values (Pearson = −0.252).

Also, a negative correlation is highlighted between tissue values of BDNF, desire, satisfaction, and pain. The correlation between tissue BDNF and desire is shown graphically in Figure 2.

**Figure 3:**
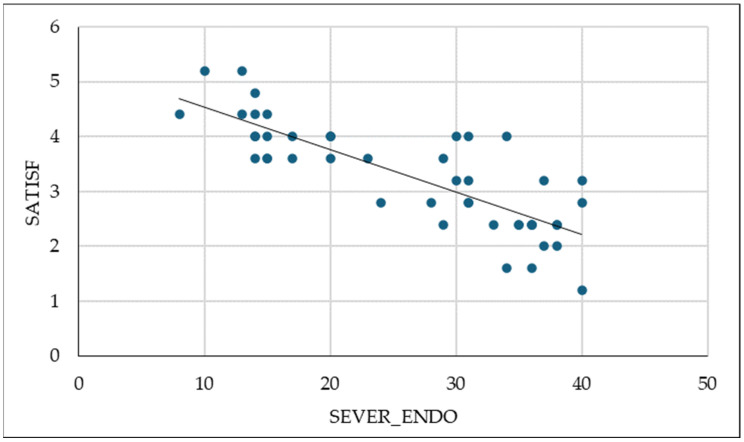
The negative association between SEVER_ENDO and sexual satisfaction values (Pearson = −0.813).

As expected, we find a significant negative correlation between the severity of endometriosis, sexual satisfaction, pain, and desire. The correlation between the severity of endometriosis and satisfaction is shown graphically in Figure 3.

**Figure 4:**
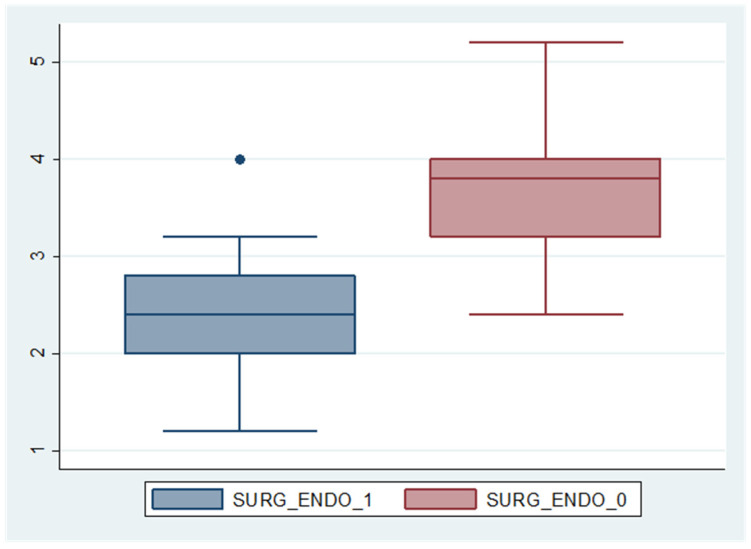
Lower average values for sexual satisfaction variable in patients who have undergone surgical interventions (variable SURG_ENDO = 1 = personal history of surgical interventions) compared to others (*p* < 0.001).

Another result obtained from the analysis is the correlation between a history of surgical interventions and the satisfaction domain of the FSFI. Patients with a history of surgical interventions for endometriosis have lower average values for the FSFI satisfaction score, as shown in Figure 4.

**Figure 5:**
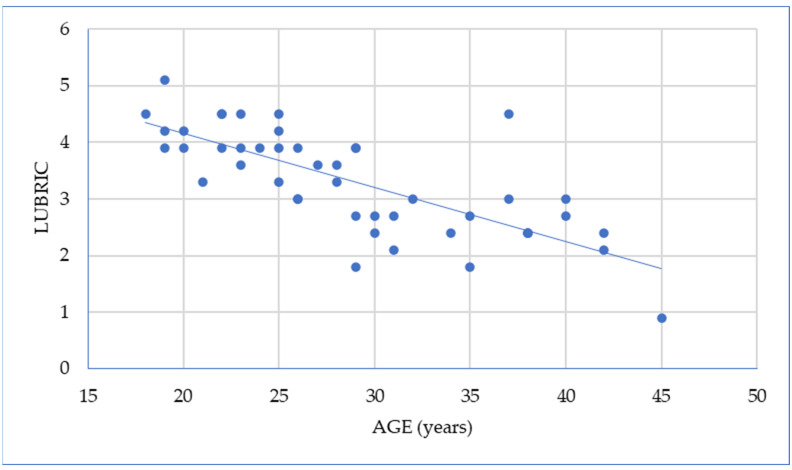
Negative association between age and lubrication values (Pearson = −0.749).

Advanced age is associated with complex hormonal changes that impact pelvic tissue structure and vascularization at this level, thereby influencing FSFI components. This relationship is demonstrated in Figure 5, where there is an inverse correlation between patient age and the lubrication domain score of the FSFI.

**Figure 6:**
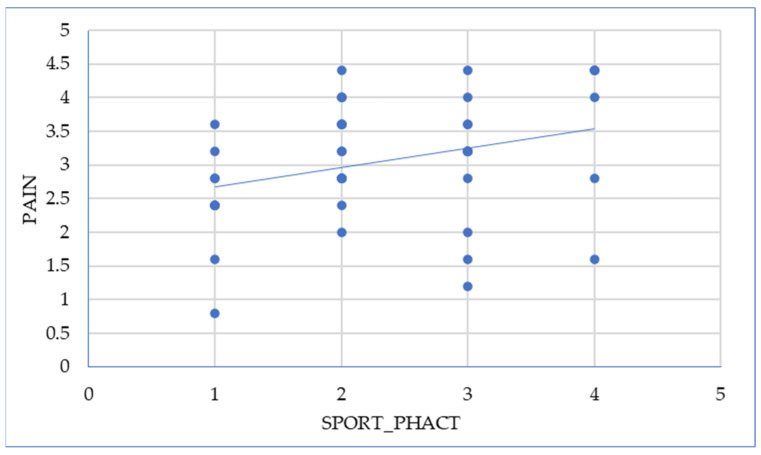
Association between sport and pain (Pearson = 0.312).

Engaging in sports improves sexual quality of life in patients with endometriosis concerning the pain domain of the FSFI; this relationship is illustrated in Figure 6.

## 3. Discussion

The quality of a patient’s sexual life should be assessed in more detail than simply evaluating pain during sexual intercourse. Dyspareunia is indeed one of the primary symptoms associated with endometriosis, especially in advanced stages or infiltrative forms affecting uterosacral ligaments, the rectovaginal septum, or the Douglas pouch. Assessing sexual dysfunction using the FSFI questionnaire allows for a detailed examination of sexual dysfunctions across several primary components: desire, arousal, pain, lubrication, and orgasm. The “Desire” domain reflects the patient’s motivation to engage in sexual relations with their partner. At the same time, patients affected by endometriosis are primarily young and of reproductive age, and it would be expected that the FSFI score would be high. The “Arousal” score follows that of desire, serving as a consequence of the first. However, it can be influenced by previous experiences. Regarding the “Lubrication” domain, one could expect it to be the least related to endometriosis, as it primarily reflects appropriate hormonal status and is directly affected by the patient’s age or certain hormonal suppression treatments. The “Pain” domain has received the most interest in studies over the last few decades because its impairment can negatively influence all other domains, lowering their scores or, more severely, leading to the patient’s avoidance of sexual intercourse with their life partner [31,36,37,38,39].

Regarding the selected biomarker panel, it targets the main mechanisms involved in the development of endometriosis: IL-8 levels can be useful for identifying inflammatory and angiogenic activity in endometriosis [40,41,42,43]; IL-10 is a key element in modulating the immune system, thereby highlighting the role of immune deficiency in the development of endometriotic lesions [44,45,46,47]; the role of BDNF in nerve sensitization and pain perception is well known and studied, with modulation of its receptor potentially becoming part of the therapeutic arsenal in personalized therapy in the future [48,49,50,51,52].

In our study, we found a negative correlation between IL-8 and the “Desire” domain of the FSFI; the higher the level of IL-8, the lower the subjects’ level of desire. The presence of acute or chronic inflammation is mediated by the release of an impressive arsenal of cytokines at the systemic level. Studies in this area show that these cytokines can induce certain responses in the mesolimbic system, cingulate cortex, or thalamus, areas of the nervous system that are relevant in mediating sexual desire and arousal. The effect of cytokines is either direct through interactions with neuronal receptors or indirect through dopamine. Additionally, an inflammatory environment can interfere with the secretion of ovarian hormones by disrupting the pulsatile release of GnRH. This effect occurs either directly or by stimulating the release of CRH-ACTH and cortisol, which will decrease GnRH secretion through a feedback mechanism [53,54,55]. On the other hand, the association of inflammation with pain during sexual intercourse is a well-studied area and has already been demonstrated in recent decades [56,57]. Our results are similar and associate high IL-8 values with low scores for the “Pain” and “Satisfaction” domains of the FSFI.

An association between BDNF levels and sexual desire was presented by Ianitelli in 2021, with our conclusions being similar to theirs, showing an inverse relationship between BDNF values and patients’ sexual desire [58]. In the case of endometriosis, the most likely mechanism is through mediating nerve terminal development and favoring the occurrence of dyspareunia, which will lead to a decrease in sexual desire, which is also probably influenced by a history of painful sexual experiences. These data are confirmed by the correlation found in our study between BDNF and the “Pain” domain in the FSFI (statistically significant correlation with *p* < 0.001) and with the “Satisfaction” domain in the FSFI (statistically significant correlation with *p* = 0.03).

Other patient-related factors can individually modify scores of the FSFI domains. Among those studied in this paper are age, body mass index, treatment followed by the patient, severity of endometriosis, history of surgical interventions, and physical activity practiced by the patient. Inverse correlations were found between the severity of endometriosis and the FSFI scores for desire, pain, and satisfaction—higher severity scores corresponded to lower scores in these domains. The same type of correlation is evident between the history of surgical interventions and the sexual satisfaction level of the subjects. Our data are consistent with results found by other authors [59,60,61].

Frequency, duration, and intensity of sports activities and physical activities outside of sports influence the Female Sexual Function Index (FSFI) in patients with endometriosis, but the effects vary across its components. Significant positive effects were found regarding desire (*p* = 0.019), indicating that physical activity may enhance sexual desire, likely due to improved blood circulation, hormonal regulation, and stress reduction, which are known to impact libido. Lubrication (*p* = 0.033) also improved, potentially due to the same physiological benefits, such as increased blood flow to the pelvic area and improved overall cardiovascular health, which can facilitate lubrication. The reduction in pain (*p* = 0.004) suggests that physical activity may help alleviate discomfort during intercourse by reducing inflammation, enhancing muscle flexibility, and improving pain tolerance, which is critical for patients with endometriosis. However, effects on arousal, orgasm, and satisfaction were not statistically significant. This may be because these domains are more complex and influenced by psychological, emotional, and relational factors beyond just physical health. For example, arousal and orgasm often require more nuanced neurophysiological stimulation, which physical activity alone may not directly address. Satisfaction is highly subjective and can be influenced by the quality of the relationship or emotional connection, areas that physical activity might not impact as strongly. Therefore, while physical activity shows clear benefits in certain physiological aspects, its effects on these more complex dimensions of sexual function may require additional psychological or emotional interventions.

Similar results about endometriosis and serum chemokines were obtained by Wojciechowska, who used patients with ovarian teratomas as a control group and studied chemokines such as MCP-1 and MCP-3 [62].

Most correlations between investigated factors (biomarkers or individual factors) were found among the “pain”, “satisfaction”, and “desire” domains of the FSFI. These results are explained by the interdependence and bidirectional influence that these domains have within the score. It is expected that a patient experiencing pain during sexual intercourse will have a reduced level of satisfaction, which will lead to a decrease in sexual desire in the future. However, many studies in the field have also shown other mechanisms through which changes in the molecular profile in endometriosis can alter sexual function, such as responses from the central nuclei involved in sexual function, alterations in functioning of the hypothalamic–pituitary–ovarian axis, or others.

However, the current study presents a series of limitations and weak points. The limited sample size in this study may affect the generalizability of the findings. With a relatively small group of participants, the results may not fully represent the broader population or account for potential variations across different demographic groups. Another limitation is the absence of a control group, which restricts the ability to conclusively attribute observed changes solely to the intervention.

The study’s duration is another limitation. The data were collected over a two-year period, which may not be sufficient to observe the long-term effects or sustainability of the intervention’s impact. A longer timeframe could provide more information on whether the observed outcomes are stable over time or if they diminish. Consequently, conclusions about the intervention’s long-term effectiveness should be considered with caution. Since this study was conducted within a specific geographic and cultural context, its findings may not be fully generalizable to other regions or cultural groups. Differences in social, economic, and cultural backgrounds can influence the behaviors and outcomes being studied, which means that applying these results universally may overlook important regional variations. Therefore, further studies in diverse contexts are recommended to enhance the applicability of these findings on a broader scale.

## 4. Materials and Methods

The study design is a cross-sectional, prospective case–control type. For this purpose, we included a group of 46 patients diagnosed with endometriosis through clinical examination and imaging and confirmed by histopathological examination, recruited in a 2-year interval between 2022 and 2023. Informed consent was obtained from each patient before inclusion in the study. Additionally, a General Data Protection Regulation (GDPR) form was signed by all subjects. The ethical and medical deontology requirements were followed in accordance with the Declaration of Helsinki, and the study was approved by the Ethics Committee of the “Iuliu Hațieganu” University of Medicine and Pharmacy, Cluj-Napoca, Romania (AVZ251 dated 25 February 2022).

Inclusion criteria: patients of reproductive age, diagnosed with histopathologically confirmed endometriosis, patients who have undergone surgical treatment for endometriosis, and patients who freely expressed their informed consent to participate in this study.

Exclusion criteria: patients who did not express informed consent for enrollment in the study or did not meet the conditions described above.

Each patient completed three forms and questionnaires:

- A questionnaire collecting general data (age, weight, weight, body mass index, background/environment, family medical history, personal medical history, personal history, and symptomatology information;
- The FSFI questionnaire;
- A questionnaire regarding the physical activity practiced by the patient.

Sexual quality of life was assessed using the Female Sexual Function Index (FSFI) questionnaire. The analysis was conducted for each of the FSFI domains and the included biomarkers. Additionally, other factors that may influence FSFI scores were analyzed, such as hormonal treatment followed by the patient, severity of endometriosis, history of surgical interventions for endometriosis, VAS (Visual Analogue Scale) scale for assessing general quality of life, age, body mass index, and physical activity practiced by the patient.

To determine serum biomarkers, a blood sample was collected from each patient before surgical intervention to analyze the serum levels of certain biomarkers. Each sample was centrifuged for 15 min at 1000× *g* at a temperature between 2 and 8 °C. The obtained supernatant was stored at −80 °C until processing. Additionally, an intraoperative tissue sample was obtained in order to analyze the tissue levels of the same biomarkers. Tissue samples were preserved in saline solution and frozen at −80 °C until processing.

Assessment of sexual quality and function in patients was carried out using the FSFI questionnaire, a comprehensive tool specifically designed to evaluate female sexual health. This questionnaire comprises 19 questions that are divided into five domains, each addressing a key component of female sexual function: sexual desire, arousal, orgasm, pain, and satisfaction. For patients who are sexually inactive, a score of 0 is assigned.

Each of the 19 questions employs a 5-point Likert scale, with responses ranging from 1 (indicating the lowest level of function) to 5 (indicating the highest level of function). The scores for questions within each domain are summed and adjusted using a specific domain weight to ensure an accurate representation of that domain’s contribution to overall sexual function. The domain-specific weights are as follows: 0.6 for desire, 0.3 for arousal, 0.3 for lubrication, 0.4 for orgasm, 0.4 for satisfaction, and 0.4 for pain.

The final domain scores are then combined to calculate the overall FSFI score, which ranges from a minimum of 2.0, indicating severe dysfunction, to a maximum of 36.0, representing optimal sexual function. This structured scoring system allows for a detailed and nuanced assessment of various aspects of sexual health, making the FSFI an essential tool in both clinical and research contexts [36].

Staging of endometriosis was performed in accordance with the clinic’s internal protocol using the rASRM classification.

### 4.1. Analysis of Investigated Biomarkers

For each patient, serum and tissue levels of the following biomarkers were measured: IL-8, IL-10, and BDNF. The analyses were conducted using specific ELISA kits from Elabscience. For IL-8, the Human IL-8 (Interleukin 8) ELISA kit (catalog no: E-EL-H6008) was used. IL-10 levels were assessed with the Human IL-10 (Interleukin 10) ELISA kit (catalog no: E-EL-H6154). BDNF levels were measured using the Human BDNF (Brain-Derived Neurotrophic Factor) ELISA kit (catalog no: E-EL-H0010). ELISA (Enzyme-Linked Immunosorbent Assay) was used for all samples. The ELISA technique is a widely used analytical method for detecting and quantifying specific proteins, antibodies, or antigens in a sample. The technique is based on the specific binding between an antigen and an antibody, with an enzyme-linked detection system that produces a measurable signal, typically a color change. ELISA is highly sensitive, specific, and versatile, making it a key tool in diagnostics, research, and various applications in immunology and biochemistry.

### 4.2. Method of Analysis

Correlations were analyzed using Student’s *t*-test for independent samples with unequal variances. Box plots were utilized to provide a clear graphical representation of data distribution. To assess the effects of various factors on FSFI domains, Ordinary Least Squares (OLS) linear regression was employed. To reduce the risk of Omitted Variable Bias (OVB) in the regression, several control variables were incorporated.

The study prioritized reliability by evaluating whether IL-8 and IL-10 retained their sign and statistical significance in regression models, both with and without the inclusion of other biomarkers. This approach was designed to address potential confounding effects associated with tissue-derived IL-8 and IL-10. Pearson correlations between FSFI domains and influencing factors were also calculated as a robustness measure. Scatter plots were used to visually examine the linearity of these correlations, further supporting the validity of the findings.

Our study employed an exhaustive sampling method, including all eligible patients diagnosed with endometriosis during the study period, ensuring comprehensive population representation and minimizing selection bias. The inclusion of 46 patients provided sufficient variability in the data, enabling robust statistical analyses that yielded significant correlations and supported the validity of our hypotheses. Specifically, the observed statistical power was adequate to detect meaningful relationships between biomarkers and clinical outcomes, as evidenced by consistent and significant findings across multiple analyses. Thus, the sample size was methodologically appropriate to achieve the study’s objectives and ensure reliable and interpretable results.

## 5. Conclusions

Sexual function is a vital component of quality of life for patients affected by endometriosis. Assessing sexual dysfunction necessitates a comprehensive approach beyond merely evaluating dyspareunia. FSFI questionnaire effectively examines the primary components of sexual function, including desire, arousal, lubrication, orgasm, satisfaction, and pain.

Alterations in the molecular profile of IL-8, BDNF, and IL-10 provide insights into the mechanisms underlying sexual dysfunction in these patients. Notably, the most significant changes were observed in the pain, satisfaction, and desire domains, with strong negative correlations identified with IL-8 and BDNF, respectively. Furthermore, the severity of endometriosis and a history of surgical interventions significantly influenced pain scores and level of sexual satisfaction, while increasing age was associated with a decrease in lubrication.

This is one of few studies that includes molecular biomarkers in the analysis of female sexual dysfunction in correlation with endometriosis, which could explain the mechanisms behind the development of this dysfunction. The report considers both the included biomarkers and other well-known factors that negatively influence sexual function: age, severity of endometriosis, BMI, and history of surgical interventions.

Future studies regarding the pathophysiological mechanisms, early diagnosis, and personalized treatment of patients with endometriosis would give a deeper understanding of mechanisms contributing to sexual dysfunction, and can facilitate effective interventions, ultimately enhancing the quality of life for affected individuals in the future.

## Figures and Tables

**Table 1:** Results of the FSFI questionnaire applied to patients.

Variable	Explanations	Mean	Std. Dev. ^$^	Min	Max
DESIRE	FSFI component, measured using Likert scale (1–5). In the FSFI component, the share of this component is 0.6	3.38	0.98	1.2	5.4
AROUSAL	FSFI component, measured using Likert scale (1–5). In the FSFI component, the share of this component is 0.3	3.08	0.52	1.8	4.2
LUBRIC	FSFI component, measured using Likert scale (1–5). In the FSFI component, the share of this component is 0.3	3.31	0.92	0.9	5.1
ORGASM	FSFI component, measured using Likert scale (1–5). In the FSFI component, the share of this component is 0.4	3.25	0.76	1.6	4.8
SATISF	FSFI component, measured using Likert scale (1–5). In the FSFI component, the share of this component is 0.4	3.28	0.96	1.2	5.2
PAIN	FSFI component, measured using Likert scale (1–5). In the FSFI component, the share of this component is 0.4	3.07	0.90	0.8	4.4

**^$^** Std. dev. = standard deviation. Source: authors’ calculations in STATA.

**Table 2:** Expression of biomarkers at serum (S) and tissue level (T).

	IL 8 S ^2^	IL 8 T ^3^	IL 10 S	IL 10 T	BDNF S	BDNF T
average	37.85	114.36	34.77	71.88	784.60	53.80
Std. dev. ^1^	36.93	39.5	20.75	20.69	505.77	25.32

^1^ standard deviation, ^2^ serum levels, ^3^ tissue levels.

**Table 3:** Associated factors that may influence FSFI score.

Variable	Explanations	Mean	Std. Dev. ^$^	Min	Max
HORMONAL	Presence or absence of hormonal treatment	0.61	0.49	0	1
SEVER_ENDO	Endometriosis severity score assessed uses the rASRM classification	26.3	9.95	8	40
SURG_ENDO	History of surgery for endometriosis, 1 if yes, 0 if not	0.35	0.48	0	1
HEALTH_VAS	Life Quality—Visual Analogue Scale 0–100	77.1	11.1	30	90
AGE	Age of the patient, in years	28.85	7.14	18	45
BMI	Body mass index, a measure that uses height and weight to estimate a person’s body fat	25.31	4.71	17.57	37.4
SPORT_PHACT	The ordinal variable on a scale from 1 to 4 that measures the frequency, intensity, and duration of practicing sports or other physical activities. Values declared by the patient	2.33	0.96	1	4

**^$^** Std. dev. = standard deviation. Source: authors’ calculations in STATA.

**Table 4:** Correlation between FSFI components, studied biomarkers, and other individual factors related to endometriosis.

	Dependent Variable					
	DESIRE	AROUSAL	LUBRIC	ORGASM	SATISF	PAIN
IL8_S	−0.008 (0.134)	−0.002 (0.751)	0.0005 (0.946)	0.008 (0.392)	0.005 (0.262)	−0.002 (0.733)
IL8_T	** −0.010 (0.013)	0.006 (0.193)	0.006 (0.226)	0.0008 (0.902)	** −0.008 (0.022)	** −0.009 (0.016)
IL10_S	0.003 (0.716)	−0.005 (0.596)	−0.005 (0.674)	−0.003 (0.852)	−0.010 (0.188)	−0.002 (0.764)
IL10_T	0.006 (0.211	−0.005 (0.325)	−0.003 (0.561)	−0.006 (0.460)	0.003 (0.497)	0.0006 (0.879)
BDNF_S	−0.0002 (0.122)	−0.0002 (0.386)	0.0002 (0.368)	0.0003 (0.304)	*** −0.0005 (0.003)	0.0001 (0.744)
BDNF_T	*** −0.0102 (0.002)	−0.006 (0.109)	0.001 (0.741)	−0.008 (0.132)	*** −0.010 (0.001)	*** −0.009 (0.03)
HORMONAL	0.275 (0.074)	0.099 (0.576)	** 0.473 (0.022)	0.101 (0.705)	** 0.276 (0.048)	** 0.357 (0.013)
SEVER_ENDO	** −0.033 (0.033)	−0.029 (0.112)	−0.027 (0.183)	0.028 (0.289)	*** −0.038 (0.009)	** −0.035 (0.014)
SURG_ENDO	0.072 (0.707)	−0.072 (0.749)	−0.313 (0.220)	−0.067 (0.843)	** −0.434 (0.016)	** −0.435 (0.017)
HEALTH_VAS	−0.006 (0.465)	−0.017 (0.099)	−0.006 (0.612)	0.014 (0.367)	0.014 (0.095)	−0.007 (0.355)
AGE	** −0.036 (0.027)	0.0002 (0.999)	** −0.052 (0.017)	0.005 (0.868)	−0.028 (0.055)	0.023 (0.115)
BMI	0.013 (0.464)	−0.007 (0.729)	−0.003 (0.896)	−0.010 (0.744)	0.012 (0.423)	0.012 (0.436)
SPORT_PHACT	** 0.226 (0.019)	0.070 (0.515)	** 0.267 (0.033)	0.179 (0.271)	0.147 (0.082)	*** 0.259 (0.004)
constant	*** 6.332 (0.000)	*** 5.525 (0.000)	*** 4.684 (0.001)	1.256 (0.491)	4.978 (0.000)	4.457 (0.000)

***, ** significant at 1%, 5%; source: own calculations in STATA 16.

## Data Availability

The data presented in this study are available on request from the corresponding author. The data are not publicly available due to internal rules.
